# The "Treatment" of Measles by Nurses

**Published:** 1898-02-05

**Authors:** 


### THE "TREATMENT" OF MEASLES BY NURSES.

In regard to the proposal, recently made by a medical officer of health, that sanitary authorities should employ nurses to visit in districts where measles prevails, "for the purpose of teaching the mothers how to treat the children," we have received the following communication from the Local Government Board:—

". . . It may be perhaps sufficient for you to know that this Board do not sanction expenditure involved in the appointments to deal with people in individual houses; unless, indeed, under circumstances which are altogether exceptional and of great emergency. They hold that a sanitary authority being for the prevention, and not the treatment or cure of disease, should only appoint nurses when, in connection with such prevention of disease, they have provided isolation hospitals where a staff of nurses is requisite."

Referring to the same subject, the *Practitioner* for February says: "We are not altogether ripe here for the 'nursing professor,' but we already have what may be called the 'nursing practitioner.' When a prize fighter is 'knocked out,' it appears to be thought the right thing to have him 'professionally' attended by a nurse. A well-known medical officer of health has quite recently delivered himself of the opinion that the medical treatment of whooping-cough and measles 'is, in ordinary cases, a mere matter of routine which any intelligent woman could with a little training undertake.' . . . But it strikes me as rather a dangerous doctrine that the treatment of even the least of the ills that flesh is heir to is 'a mere matter of routine.' It is just possible that the General Medical Council might be invited to consider the position of a medical officer who should officially sanction the employment of unqualified persons—however 'intelligent' and 'well-trained'—to undertake the medical treatment of measles and whooping-cough." This is a most reasonable view of a most uncalled-for innovation.

